# Determinants of cigarette and e-cigarette use among youth and young adults-PolNicoYouth study results

**DOI:** 10.1093/eurpub/ckac130.155

**Published:** 2022-10-25

**Authors:** I Wężyk-Caba, M Znyk, R Zajdel, L Balwicki, A Tyrańska-Fobke, G Juszczyk, B Świątkowska, K Zajdel, D Kaleta

**Affiliations:** 1 Department of Hygiene and Epidemiology, Medical University of Lodz, Lodz, Poland; 2 Department of Business and Informatics, University of Łódź, Lodz, Poland; 3 Department of Public Health and Social Medicine, Medical University of Gdańsk, Gdansk, Poland; 4 Department of Public Health, Medical University of Warsaw, Warsaw, Poland; 5 Department of Environmental Epidemiology, Occupational Medicine Institute, Lodz, Poland; 6 Department of Medical Informatics and Statistics, Medical University of Lodz, Lodz, Poland

## Abstract

Identifying predictors of e-cigarette use initiation is important for preventing young persons from becoming smokers. Because of the addictiveness, harmful effects but on the other hand attractiveness and fashion for e-cigarettes among young people, teen use of tobacco related products is a significant public health concern. This study evaluated the determinants of susceptibility to e-cigarette use to both e-cigarettes use and traditional cigarettes in secondary school students in Poland. This study examined a sample of Polish youths aged 13-19 (n = 19241) attending 192 schools, 12 on average in each voivodship. Logistic regression and multivariable logistic regression models were used to calculate crude and adjusted odds ratios. The profile of susceptibility to e-cigarettes use among never e-cigarette users included: pocket money available per month (more than 150 PLN) (OR = 1.7; p = 0.001), 16-17 years old (OR = 1.9; p = 0.001), parental tobacco smoking and e-cigarette usage (OR = 2.0; p = 0.01 and OR = 1.7; p = 0.001 respectively), maternal secondary education (OR = 1.1; p = 0.04) and living in big cities >500 thou. inhabitants (OR = 1.4; p = 0.04). E-cigarette susceptible persons among ever users were similar to never cigarette users in their opinion that e-cigarettes use are less harmful than conventional smoking (OR = 1.6; p = 0.0012) and living with both parents smoking cigarettes (OR = 1.3; p = 0.02). Additionally, the determinants were: female gender (OR = 1.5; p = 0.009) in the age group less than 15 years of age (OR = 1.3; p = 0.007). The results revealed that such basic predictors as: parental smoking and opinion of lower harmfulness of e-cigarettes use are the most important determinants of smoking susceptibility among never or ever e-cigarette users. The intervention should be focused on educating the young people and their parents on the addictiveness and harmful effects of e-cigarette use and smoking.

**Key messages:**

- Such basic predictors as: parental smoking and opinion of lower harmfulness of e-cigarettes use are the most important determinants of smoking susceptibility among never or ever e-cigarette users.
- The intervention should be focused on educating the young people and their parents on the addictiveness and harmful effects of e-cigarette use and smoking.

